# Autoimmune hearing loss and ulcerative colitis

**DOI:** 10.1016/S1808-8694(15)31007-7

**Published:** 2015-10-19

**Authors:** Renato Prescinotto, Raul Vitor Rossi Zanini, Priscila Bogar Rapoport, Carlos Augusto Anadão, Renata Ribeiro de Mendonça, Roberta Borges Novais Petrilli

**Affiliations:** a MD, Otorhinolaryngology Resident - Medical School of the ABC; b MD, Otorhinolaryngology Resident - Medical School of the ABC; c MD, Otorhinolaryngologist, PhD in Otorhinolaryngology - FMUSP. Full Professor of Otorhinolaryngology - Medical School of the ABC; d MD, Otorhinolaryngologist, Assistant Professor of Otorhinolaryngology - Medical School of the ABC; e MD, Otorhinolaryngology Resident - Medical School of the ABC; f MD, Otorhinolaryngology Resident - Medical School of the ABC

**Keywords:** hearing loss, autoimmune diseases, colitis, ulcerative

## INTRODUCTION

McCabe first described autoimmune hearing loss in 1979^1^.

Summers and Harker described the first case of dysacusis associated with ulcerative colitis in 1982. Since then other cases have been reported^2^, ^3^.

## CASE PRESENTATION

This is a male 23-year-old patient presenting with right tinnitus, ipsilateral hypoacusis and dizziness following three months of diarrhea and weight loss. The patient was taking deflazacort and budesonide, which improved his intestinal, hearing and postural symptoms, but not the tinnitus.

Initial audiometry revealed mild right sensorineural dysacusis.

Laboratory exams included a positive ANF, an increased C-reactive protein, mucoproteins and HSV. Otoneurology suggested a right peripheral vestibular deficit syndrome. Magnetic resonance imaging was normal.

Colonoscopy showed colon and rectal hyperemia with erosions and ulcers, distal transverse colon and distal rectal stenosis; pathology disclosed non-specific chronic rectocolitis.

**Figure 1:**
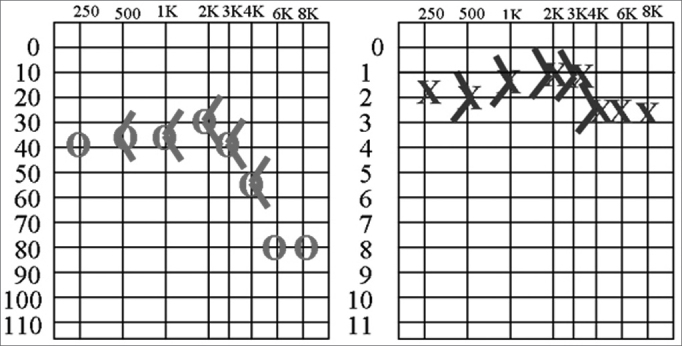
Pure tone and vocal audiometry during recurrence of the condition.

The patient used medication during one month and remained clinically stable for a further three months before recurring. Audiometry revealed a right auditory decline in all frequencies, mostly for higher frequency sounds. (Figure 1)

Prednisone (60 mg per day) was introduced, with clinical improvement, after which the dose was reduced to 20 mg per day. Follow-up audiometry revealed audiometric improvement, especially at 1 and 2KHz. Mild asymptomatic left dysacusis for lower frequency sounds was also observed.

The patient remained with occasional right hypoacusis and constant tinnitus, although this did not interfere in his daily activities; intestinal symptoms also regressed. At present the patient is taking mesalazine and prednisone.

## DISCUSSION

Sensorineural hearing loss has frequently been described in association with a variety of autoimmune diseases^3^, ^4^, ^5^, ^6^.

Although the pathophysiology of sensorineural deafness remains obscure, the autoimmune nature of the disease strengthens some proposed theories that explain its appearance and progression^1^, ^2^, ^3^, ^4^, ^5^, ^6^.

These forms of dysacusis usually present as a subclinical condition or as severe bilateral hearing loss, such as sudden deafness^5^.

There are no specific tests to establish the diagnosis of autoimmune dysacusis. A few proteins are being isolated and may have a strong connection with the autoimmune condition, such as the HSP 70 protein, although these findings are of little practical value at present^5^, ^6^.

Treatment includes aggressive use of immunosuppressants, usually corticosteroids. Other immunosuppressants have also been suggested by some authors^3^, ^4^, ^5^.

The differential diagnosis is required for cases of sensorineural dysacusis. However, exams will not necessarily confirm a specific etiology. In such cases a treatment may be given as a test to confirm the diagnosis.

## FINAL COMMENTS

Autoimmune dysacusis is one of the few sensorineural conditions in which clinical improvement may be obtained, as long as the diagnosis is promptly made and treatment is adequately employed.
